# A comparative study on the clinical efficacy of percutaneous transforaminal endoscopic discectomy versus fenestration discectomy for single-level lumbar disc herniation

**DOI:** 10.3389/fsurg.2026.1844626

**Published:** 2026-08-17

**Authors:** Jie Ma, Hengjun Wang

**Affiliations:** 1 Department of Spine Surgery, Bozhou District People's Hospital, Zunyi, Guizhou, China; 2 Guizhou Province Zunyi City Bozhou District Internet Professionals Association, Guizhou, China

**Keywords:** discectomy, herniation, endoscopic, lumbar, outcomes

## Abstract

**Objective:**

To compare the clinical outcomes of percutaneous transforaminal endoscopic discectomy (PTED) with traditional fenestration discectomy (FD) for single-level lumbar disc herniation (LDH).

**Methods:**

We retrospectively reviewed 368 patients with single-level LDH treated surgically between January 2019 and December 2021. Propensity score matching (PSM) was used to create balanced groups (*n* = 150 per group). Outcomes included clinical effectiveness (modified MacNab criteria), functional disability (ODI), pain severity (VAS), perioperative parameters, complications, and recurrence rates at 12 months. Analysis of covariance (ANCOVA) adjusting for preoperative scores was performed for primary functional outcomes.

**Results:**

Clinical effectiveness at 12 months was higher in the PTED group (92.67% vs. 80.00%, *P* < 0.001). After adjusting for preoperative values, ODI scores at 12 months remained significantly lower in PTED patients (12.4 ± 5.1 vs. 25.8 ± 8.3, adjusted *P* < 0.001), as were VAS scores (1.1 ± 0.7 vs. 2.7 ± 1.0, adjusted *P* < 0.001). The PTED group showed reduced blood loss (55.8 ± 26.3 mL vs. 88.3 ± 39.4 mL, *P* < 0.001), shorter hospitalisation (1.5 ± 0.6 vs. 5.5 ± 0.6 days, *P* < 0.001), and lower complication rates (2.67% vs. 13.33%, *P* = 0.001). Recurrence at 12 months was 2.67% in PTED versus 8.67% in FD (*P* = 0.025).

**Conclusion:**

PTED offers favourable short-term clinical outcomes compared with open FD for single-level LDH, with less perioperative morbidity and lower early recurrence rates. Longer follow-up is needed to confirm durability of these results.

## 1 Introduction

Lumbar disc herniation (LDH) is among the most common spinal disorders encountered in clinical practice, affecting approximately 1%–3% of the general population and representing a major cause of disability in working-age adults (1, 2). When conservative treatment fails to provide adequate relief, surgical intervention becomes necessary in roughly 10%–15% of patients (3).

Open fenestration discectomy (FD) has been the standard surgical treatment for decades, providing reliable neural decompression (4). However, it requires paraspinal muscle dissection and partial laminectomy, which may lead to postoperative instability, epidural fibrosis, and prolonged recovery (5, 6). Percutaneous transforaminal endoscopic discectomy (PTED) has gained popularity as a minimally invasive alternative that accesses the disc through a posterolateral approach under local anaesthesia, theoretically preserving posterior spinal structures (7, 8).

Although several studies have compared endoscopic and open techniques, most are limited by small sample sizes, short follow-up, or inadequate control of confounding variables (9, 10). The present study aimed to compare clinical outcomes between PTED and FD in a propensity-score-matched cohort with 12-month follow-up, including covariate-adjusted analyses and detailed reporting of herniation characteristics.

## 2 Methods

### 2.1 Ethical approval

This study was approved by the Ethics Committee of Bozhou District People's Hospital (Approval No. BZPH-2022-IRB-037). Given the retrospective nature of the study, the requirement for individual written informed consent was waived by the ethics committee. All procedures performed in this study were in accordance with the 1964 Helsinki Declaration and its subsequent amendments. Patient data were de-identified prior to analysis.

### 2.2 Study design and patient selection

This single-centre retrospective comparative study was conducted at the Department of Spine Surgery, Bozhou District People's Hospital. We reviewed consecutive patients who underwent surgery for single-level LDH between January 2019 and December 2021. The initial cohort comprised 368 patients: 216 underwent PTED and 152 underwent FD. Inclusion criteria were: (1) age 18–75 years; (2) MRI-confirmed single-level LDH; (3) radicular symptoms persisting despite ≥6 weeks of conservative treatment; (4) concordance between clinical presentation and imaging findings. Exclusion criteria included: multilevel disc disease, significant spinal stenosis, spondylolisthesis, prior surgery at the index level, severe osteoporosis, active infection, and malignancy.

### 2.3 Propensity score matching

To reduce selection bias, propensity score matching (PSM) was performed using a logistic regression model including the following covariates: age, sex, body mass index (BMI), symptom duration, herniation size, herniation location, herniation morphology (protrusion, extrusion, or sequestration), smoking status, diabetes, and preoperative ODI and VAS scores. One-to-one nearest-neighbour matching was conducted with a caliper width of 0.2 standard deviations of the logit of the propensity score. Covariate balance was assessed using standardised mean differences (SMD), with SMD <0.1 considered adequate. After matching, 150 patients remained in each group.

### 2.4 Surgical procedures

PTED: Patients were positioned prone on a radiolucent table. Under local anaesthesia with intravenous sedation, a posterolateral approach was used to access the target foramen through a 7–8 mm skin incision. A working cannula was placed under fluoroscopic guidance, and disc material was removed under direct endoscopic visualisation (rigid endoscope, outer diameter 6.9 mm, working channel 3.7 mm). Three spine surgeons performed all PTED procedures (mean endoscopic experience: 8.5 ± 1.8 years; each had completed >200 PTED cases prior to the study period).

Fenestration discectomy: Under general anaesthesia, a midline posterior approach was used through a 3–4 cm incision. After subperiosteal muscle dissection, a partial laminotomy and ligamentum flavum resection were performed to expose the nerve root and disc. Herniated material was removed under direct visualisation. Five spine surgeons performed FD procedures (mean open surgery experience: 12.3 ± 2.1 years).

### 2.5 Surgeon experience assessment

To evaluate potential performance bias, we calculated intraclass correlation coefficients (ICC) for primary outcomes among surgeons within each group. ICC values were 0.08 (95% CI: 0.00–0.24) for PTED surgeons and 0.06 (95% CI: 0.00–0.19) for FD surgeons, indicating negligible inter-surgeon variability in outcomes within each technique.

### 2.6 Outcome measures

The primary outcome was clinical effectiveness at 12 months, assessed using modified MacNab criteria (excellent + good = effective). Secondary outcomes included ODI (0–100), VAS for leg pain (0–10), SF-36 physical component summary, patient satisfaction (0–10 numeric rating scale), return-to-work status, perioperative parameters (operative time, blood loss, hospital stay, time to ambulation), complications (classified by Clavien-Dindo grade), and recurrence (defined as same-level reherniation confirmed by MRI requiring reoperation). Outcomes were assessed at 1, 3, 6, and 12 months postoperatively.

### 2.7 Statistical analysis

Continuous variables are presented as mean ± SD and compared using independent-samples t-tests. Categorical variables are presented as frequencies and compared using chi-square or Fisher's exact tests. For primary functional outcomes (ODI, VAS), analysis of covariance (ANCOVA) was performed with preoperative scores as covariates to adjust for any residual baseline differences after PSM. Repeated-measures ANOVA was used to assess time-by-group interactions across follow-up time points; assumptions of sphericity (Mauchly's test) and normality (Shapiro–Wilk) were verified. Kaplan–Meier analysis with log-rank testing was used for recurrence-free survival. Cox proportional hazards regression was used to estimate hazard ratios. Multivariate logistic regression identified independent predictors of recurrence. Subgroup analyses were performed stratified by age, BMI, symptom duration, and herniation size; Bonferroni correction was applied (adjusted *α*=0.0125 for 4 subgroups). Effect sizes were reported as Cohen's d. All analyses were two-tailed with *α*=0.05. SPSS 26.0 (IBM Corp.) was used for all analyses.

## 3 Results

### 3.1 Baseline characteristics

After PSM, 150 patients were included in each group. Baseline demographics, clinical characteristics, and herniation parameters were well balanced (all SMD <0.1; Table 1). Preoperative ODI and VAS scores were comparable between groups.

**Table 1:** Baseline characteristics after propensity score matching.

Characteristic	PTED (*n* = 150)	FD (*n* = 150)	*P* value	SMD
Age (years)	45.3 ± 11.2	46.1 ± 10.8	0.524	0.07
Sex (male/female)	89/61	91/59	0.762	0.03
BMI (kg/m²)	25.4 ± 3.1	25.7 ± 3.3	0.412	0.09
Symptom duration (months)	8.2 ± 4.5	8.5 ± 4.8	0.638	0.06
Smoking status (yes/no)	52/98	54/96	0.798	0.03
Diabetes (yes/no)	18/132	20/130	0.728	0.04
Preoperative ODI	62.4 ± 12.8	63.1 ± 13.2	0.642	0.05
Preoperative VAS	7.2 ± 1.3	7.3 ± 1.4	0.518	0.07
Herniation size (small/medium/large)	45/78/27	48/75/27	0.892	0.05
Herniation location (central/paracentral/lateral/foraminal)	32/68/35/15	35/66/36/13	0.756	0.08
Herniation morphology (protrusion/extrusion/sequestration)	62/71/17	65/68/17	0.864	0.04
Occupation (manual/sedentary/mixed)	52/61/37	54/59/37	0.834	0.03
Neurological deficit (yes/no)	43/107	45/105	0.778	0.03

### 3.2 Perioperative outcomes

Perioperative data are summarised in Table 2. Operative time was comparable between groups. The PTED group had significantly less intraoperative blood loss, shorter hospital stay, and earlier ambulation (all *P* < 0.001).

**Table 2:** Perioperative outcomes.

Parameter	PTED (*n* = 150)	FD (*n* = 150)	*P* value
Operative time (min)	68.5 ± 15.3	72.1 ± 18.7	0.071
Blood loss (mL)	55.8 ± 26.3	88.3 ± 39.4	<0.001
Hospital stay (days)	1.5 ± 0.6	5.5 ± 0.6	<0.001
Time to ambulation (days)	1.0 ± 0.2	1.4 ± 0.3	<0.001
Complication rate	4/150 (2.67%)	20/150 (13.33%)	0.001

### 3.3 Functional outcomes

Both groups showed significant improvement in ODI and VAS scores over time (*P* < 0.001 for time effect). Repeated-measures ANOVA demonstrated significant time-by-group interactions for both ODI (F = 28.6, *P* < 0.001) and VAS (F = 24.3, *P* < 0.001). ANCOVA adjusting for preoperative scores confirmed that the PTED group had significantly lower ODI and VAS at all postoperative time points (Table 3). Effect sizes at 12 months were large for both ODI (Cohen's d = 1.95) and VAS (Cohen's d = 1.85).

**Table 3:** Functional outcomes at 12 months (ANCOVA-adjusted).

Outcome	PTED (*n* = 150)	FD (*n* = 150)	*P* value
Clinical effectiveness rate (%)	139/150 (92.67%)	120/150 (80.00%)	<0.001
ODI at 12 months (adjusted)	12.4 ± 5.1	25.8 ± 8.3	<0.001
VAS at 12 months (adjusted)	1.1 ± 0.7	2.7 ± 1.0	<0.001
SF-36 PCS at 12 months	49.2 ± 4.6	42.8 ± 5.9	<0.001
Patient satisfaction (0–10)	8.9 ± 0.8	7.6 ± 1.2	<0.001
Return to work (%)	142/150 (94.67%)	123/150 (82.00%)	0.001

### 3.4 Complications

The overall complication rate was lower in the PTED group (2.67% vs. 13.33%, *P* = 0.001). In the PTED group, complications included transient dysaesthesia (*n* = 2, Grade I) and dural puncture managed conservatively (*n* = 2, Grade I). In the FD group, complications included wound infection requiring antibiotics (*n* = 5, Grade II), transient nerve root injury (*n* = 4, Grade I), symptomatic epidural haematoma requiring evacuation (*n* = 2, Grade IIIb), excessive intraoperative bleeding requiring transfusion (*n* = 3, Grade II), dural tear repaired intraoperatively (*n* = 4, Grade I), and deep venous thrombosis (*n* = 2, Grade II).

### 3.5 Recurrence

At 12-month follow-up, the recurrence rate was 2.67% (4/150) in the PTED group and 8.67% (13/150) in the FD group (*P* = 0.025). Kaplan–Meier analysis showed a significant difference in recurrence-free survival (log-rank *χ*²=5.83, *P* = 0.016). Cox regression yielded a hazard ratio of 0.30 (95% CI: 0.10–0.91, *P* = 0.033) for PTED relative to FD (Table 4).

**Table 4:** Recurrence analysis.

Parameter	PTED (*n* = 150)	FD (*n* = 150)	*P* value
Recurrence at 6 months	2/150 (1.33%)	7/150 (4.67%)	0.090
Recurrence at 12 months	4/150 (2.67%)	13/150 (8.67%)	0.025
Hazard ratio (95% CI)	0.30 (0.10–0.91)	Reference	0.033
Log-rank *χ*²	5.83	—	0.016

### 3.6 Subgroup and prognostic factor analysis

Subgroup analyses stratified by age (<40, 40–60, >60 years), BMI (<25, 25–30, >30 kg/m²), symptom duration (<6, 6–12, >12 months), and herniation size consistently favoured PTED, with no significant interaction effects (all *P*-interaction >0.10).

Multivariate logistic regression identified surgical technique (PTED vs. FD: OR 0.28, 95% CI 0.10–0.79, *P* = 0.016), symptom duration >12 months (OR 2.64, 95% CI 1.12–6.22, *P* = 0.026), and large herniation size (OR 3.18, 95% CI 1.24–8.15, *P* = 0.016) as independent predictors of recurrence. The model showed adequate calibration (Hosmer-Lemeshow *P* = 0.68) and discrimination (AUC=0.79, 95% CI: 0.69–0.89).

## 4 Discussion

The present propensity-score-matched analysis indicates that PTED provides favourable clinical outcomes compared with open FD for single-level LDH at 12-month follow-up. The PTED group showed favourable functional recovery, fewer complications, and lower recurrence rates, even after adjusting for preoperative functional status. These results should be interpreted with caution given the inherent limitations of the retrospective design.

The perioperative advantages of PTED—less blood loss, shorter hospitalisation, and earlier mobilisation—are consistent with its minimally invasive nature and are consistent with findings from previous comparative studies (9–11). The preservation of posterior musculoligamentous structures likely contributes to faster early recovery. However, the two techniques differed in anaesthetic modality (local vs. general anaesthesia), which may independently influence early recovery parameters such as time to ambulation and immediate postoperative pain. This represents an inherent difference between the two procedures as practised clinically, rather than a confounding variable *per se*, but it limits the ability to attribute all early recovery differences solely to the surgical technique.

The lower recurrence rate in the PTED group (2.67% vs. 8.67% at 12 months) deserves careful interpretation. The FD recurrence rate in our cohort is consistent with published rates of 5%–15% for open discectomy (12, 13), while the PTED rate is consistent with recent large series reporting 1%–5% (14, 15). The difference may relate to more targeted disc removal under magnified endoscopic visualisation, which allows selective removal of pathological tissue while preserving healthy annular and nuclear structures. Additionally, the minimal disruption of posterior stabilising structures with PTED may reduce abnormal segmental loading that predisposes to reherniation.

The identification of prolonged symptom duration and large herniation size as independent risk factors for recurrence is clinically relevant and consistent with prior reports (16, 17). These findings may assist in preoperative counselling and patient selection.

Compared with existing literature, this study contributes several methodological improvements. First, PSM was employed to balance a wide range of baseline covariates including herniation morphology and preoperative functional scores, which are often neglected in prior comparative studies. Second, ANCOVA adjusting for preoperative ODI and VAS provides more reliable estimates of treatment effects than unadjusted comparisons. Third, the detailed reporting of herniation characteristics and surgeon experience addresses common criticisms of earlier non-randomised comparisons.

Several limitations should be acknowledged. First, despite PSM, unmeasured confounders may persist given the retrospective design; variables such as sagittal alignment, segmental instability, and patient expectations were not captured. Second, the difference in anaesthetic technique and postoperative mobilisation protocols between groups—reflecting standard clinical practice for each procedure—may have contributed to differences in early recovery outcomes. Future prospective trials should consider standardising perioperative protocols where feasible. Third, the 12-month follow-up, while an improvement over our initial 6-month data, may still be insufficient to capture late recurrences that can occur up to 2–5 years postoperatively; continued surveillance of this cohort is ongoing. Fourth, we did not perform systematic radiological assessment of disc height or segmental stability, which would strengthen biomechanical conclusions; this was not feasible given the retrospective design but should be incorporated in future prospective studies. Fifth, this is a single-centre study, and generalisability to other settings requires confirmation. Finally, the non-randomised design cannot establish causality, and a well-designed randomised controlled trial remains the ideal next step.

## 5 Conclusion

In this propensity-score-matched retrospective study, PTED demonstrated favourable functional outcomes, fewer complications, and lower recurrence rates compared with open FD for single-level LDH at 12-month follow-up. These findings support PTED as a viable alternative to open surgery for appropriately selected patients. However, the retrospective design and relatively short follow-up preclude definitive conclusions regarding long-term superiority. Prospective randomised trials with longer follow-up are warranted to confirm these results.

## Data Availability

The raw data supporting the conclusions of this article will be made available by the authors, without undue reservation.
